# Analysis of the Correlation of Galectin-3 Concentration with the Measurements of Echocardiographic Parameters Assessing Left Atrial Remodeling and Function in Patients with Persistent Atrial Fibrillation

**DOI:** 10.3390/biom11081108

**Published:** 2021-07-28

**Authors:** Paweł Wałek, Urszula Grabowska, Elżbieta Cieśla, Janusz Sielski, Joanna Roskal-Wałek, Beata Wożakowska-Kapłon

**Affiliations:** 1 Collegium Medicum, The Jan Kochanowski University, 25-317 Kielce, Poland; urszulagrabowskaa@gmail.com (U.G.); ela66ciesla@gmail.com (E.C.); jsielski7@interia.pl (J.S.); joanna.roskal.walek@wp.pl (J.R.-W.); bw.kaplon@poczta.onet.pl (B.W.-K.); 2 1st Clinic of Cardiology and Electrotherapy, Swietokrzyskie Cardiology Centre, 25-736 Kielce, Poland

**Keywords:** atrial fibrillation, biomarkers, cardioversion, galectin-3, left atrial strain

## Abstract

Galectin-3 (gal-3) is a fibrosis marker and may play a role in fibrosis of the left atrium (LA). Left atrial wall fibrosis may influence the transition from paroxysmal to non-paroxysmal atrial fibrillation (AF). In this study, we assessed the correlation of gal-3 concentration with the main echocardio-graphic parameters evaluating dimensions, volume, compliance, and left atrial contractility during AF and after successful electrical cardioversion (DCCV). The study included 63 patients with left atrial enlargement who qualified for DCCV due to persistent AF. The procedure recovered sinus rhythm in 43 (68.3%) patients. The concentration of gal-3 was negatively correlated with the echocardiographic parameters of LA including dimensions (LA length pre, rho = −0.38; *p* = 0.003), volume (LAV pre, rho = −0.39; *p* = 0.003), compliance (LASr mean post, rho = −0.33) and contractility (pLASRct mean post, rho = −0.33; *p* = 0.038). Negative correlations of gal-3 concentration were also observed in relation to the volume and contractility of the left ventricle. The concentration of gal-3 significantly negatively correlates with the size, systolic function, and compliance of the LA wall in patients with persistent AF. Determining gal-3 concentration in patients with persistent AF may help in the assessment of remodeling of the LA wall.

## 1. Introduction

Atrial fibrillation (AF) is the most common persistent supraventricular tachyarrhythmia [1]. It is associated with an increased risk of developing heart failure, stroke and premature death [2]. The number of patients with AF in the adult population in Europe and the United States is growing. In 2010, the total number of people with AF worldwide was estimated at 20.9 million men and 12.6 million women [3,4]. According to forecasts, by 2030 the number of patients with AF in Europe will increase to 14–17 million and the annual number of new patients with such a diagnosis will be 120,000–215,000 [4,5,6]. The treatment of AF is also associated with a very heavy financial burden on the health service. According to a study by Ki et al., the increase in AF treatment costs in the USA in 2008 amounted to USD 8075 per person, which in relation to the entire population ranged between 6.0 and 26.0 trillion U.S. dollars [7]. Data for Western Europe are similar and indicate a significant escalation of costs associated with the hospitalization of patients with AF [7,8,9,10,11,12].

Many biomarkers to assess the risk of AF recurrence or thromboembolic events after direct current cardioversion (DCCV) or ablation are currently being investigated. One of the biomarkers introduced in the clinic relatively recently and used in the assessment of patients with heart failure and AF is galectin-3 (gal-3). Biochemically, it is a protein with a molecular weight of 35 KDa. It is encoded by a single gene: LGALS3, located in position q21-q22 on chromosome 14 [13,14]. Like other galectins, gal-3 is mainly present in the cytoplasm [15]. Gal-3 is found in many types of cell: macrophages, neutrophils, mast cells, fibroblasts, and osteoclasts. In other words, it is found mainly in cells involved in inflammatory and fibrotic processes [16]. Gal-3 also activates neutrophils and T lymphocytes, and regulates intercellular adhesion, angiogenesis, cell growth and differentiation, as well as apoptosis. The actions of damaging factors on the cells of the heart muscle stimulate macrophages to secrete gal-3. One of the most important pathways of gal-3’s activity is its effect on fibroblasts and collagen synthesis. Gal-3 stimulates collagen synthesis and thus contributes to the impairment of the systolic and diastolic functions of the heart muscle [17,18,19,20,21].

So far, data presented showing the prognostic value of gal-3 in the prognosis of maintaining sinus rhythm (SR) after DCCV or ablation in patients with persistent and paroxysmal AF have been inconclusive [22,23,24,25,26,27,28,29,30,31,32]. Due to the emerging reports on the possibility of treatment with galectin inhibitors, the issue of gal-3’s relationship with the pathogenesis of AF and the possibility of improving the prognosis in patients with AF seem to be of particular interest [33,34].

In this study, we present the analysis of the correlation of gal-3 levels with traditional and new echocardiographic parameters describing the degree of left atrial structural and mechanical remodeling in patients with persistent AF and left atrial enlargement qualified for DCCV.

## 2. Materials and Methods

The study protocol was approved by the Institutional Review Board of the Swietokrzyskie Medical Chamber. We included patients with persistent AF, who qualified for DCCV between February 2016 and August 2018. The inclusion criteria were as follows: symptomatic, persistent AF for 7 or more days; ejection fraction of left ventricle (LV) during AF > 50%; and appropriate anticoagulation (warfarin, acenocoumarol, dabigatran, rivaroxaban, or apixaban) for 3 or more weeks before DCCV. The exclusion criteria were: age below 18 years; no consent to participate in the study; no consent for DCCV; receiving poor quality echocardiograms; moderate or severe valve regurgitation or valve aortic stenosis; existence of valvular prosthesis; existence of thrombus in the left atrial appendage; acute myocardial infarction; acute decompensation of heart failure; a history of pulmonary vein isolation, anemia with hemoglobin of 6.9 mmol/L or less, dysthyroidism, acute infection, immune conditions, and neoplastic disease. Clinical data collection took place immediately before DCCV. Echocardiographic images were performed immediately prior to DCCV and on the next day during SR. Blood samples were drawn directly before DCCV.

### 2.1. Clinical Data

Baseline clinical assessment comprised age, sex, body mass index (BMI), smoking status, body surface area (BSA, based on Gehan and George formula), diabetes mellitus, hypertension, dyslipidemia, dysthyroidism, heart failure, coronary artery disease, history of stroke or transient ischemic attack, obstructive pulmonary disease, renal disease, EHRA (European Heart Rhythm Association) score, and data about pharmacological treatment. Diagnosis of coronary artery disease was based on a history of myocardial infarction, percutaneous coronary intervention, or aortocoronary bypass grafting. The glomerular filtration rate was calculated using the Cockcroft–Gault equation. The CHA_2_DS_2_-VASc and HAS-BLED scores were calculated as claimed by the current European guidelines on AF management [35].

All electrical DCCV were performed with anesthesiologic assistance under general anesthesia as previously described [28].

### 2.2. Echocardiographic Evaluation

Transthoracic echocardiography was performed according to current guidelines using a Vivid S6 ultrasound machine (General Electric Medical Systems, Horten, Norway) supplied with an M4S RS probe [36,37]. Transthoracic echocardiography during atrial fibrillation was performed on the day of or one day before DCCV. In patients whose SR has restored, transthoracic echocardiography was performed the day after successful DCCV. All echocardiography scans were performed by one investigator. Usual M-mode Doppler imaging and two-dimensional cine loops of parasternal long- and short-axis views and apical two-, three- and four-chamber views were obtained in every patient. All images and measurements were acquired from standard views and then digitally stored. Afterwards we analyzed images using EchoPAC PC software (GE Medical Systems). Left atrium (LA) length and transverse dimension were measured from the four-chamber view. The LA length measurement was performed perpendicularly from the mid-point of the segment that unifies the hinge points of the mitral leaflets up to the ceiling of the LA. The measurement of the LA transverse dimension was made from the lateral wall to the interatrial septum. The left atrial end-systolic and end-diastolic volumes (LAV and LAEDV) were calculated using Simpson’s method from apical four- and two-chamber views. The maximum left atrial volume (LAV) was measured at the end systole on the frame preceding mitral valve opening by tracing the left atrial inner border, with avoiding the area under the valve annulus, appendage, and pulmonary veins. LAV was indexed to BSA (LAVI). The left atrial minimum volume (LAEDV) was measured at the end of ventricular diastole, on the frame of mitral valve closure, and indexed to BSA (LAEDVI). The left atrial emptying fraction (LAEF) was calculated using the following equation:

[(left atrial maximum volume− left atrial minimum volume)/left atrial maximum volume] × 100

The left ventricular volume and ejection fraction were measured using Simpson’s method. The right atrial area was measured from the apical four-chamber view at the end systole (RAAs) and end diastole (RAAd) on the frame of tricuspid valve closure. Transmitral pulsed Doppler was recorded from the apical four-chamber view with sample volume of 2 mm placed between the tips of the mitral leaflets. Pulsed tissue Doppler imaging of the mitral annulus motion was performed from the apical four-chamber view with a 5 mm sample volume at the lateral and septal basal regions. The s’ mean, e’ mean and a’ mean were retrieved as averages from septal and lateral measurements. The measurements obtained during AF were averaged from five consecutive beats. Speckle tracking echocardiography was used to analyze left atrial deformation. The measurements of the left atrial global longitudinal strain were taken in the four-chamber and two-chamber projections. Subsequently, the average of both projections was calculated according to the recommendations of the European Association of Cardiovascular Imaging (EACVI) consensus document [38]. The left atrial wall deformation evaluated during AF was presented as the left atrial strain in the reservoir phase (LASr) and as the peak strain rate during the reservoir (pLASRr) and conduit phases (pLASRcd). Left atrial wall deformation assessed during SR after successful DCCV was presented as the left atrial strain during the reservoir phase (LASr), conduit phase (LAScd) and contraction phase (LASct), and as the peak strain rate during the reservoir phase (pLASRr), conduit phase (pLASRcd) and contraction phase (pLASRct).

### 2.3. Measurements of Galectin-3

Blood samples were drawn from the cubital vein before DCCV after overnight fasting and rest, while the patient was sitting. Following centrifugation, blood serum was snap-frozen and stored at −70 °C. The concentrations of gal-3 were determined in the samples using Quantikine Human Galectin-3 immunoassay (R&D, Systems Europe, Abingdon, Oxfordshire, UK) in August 2018. The Quantikine Human Galectin-3 Immunoassay is a 4.5 h solid phase ELISA designed to measure gal-3 levels in serum. It contains *E. coli* expressed recombinant human gal-3 and antibodies raised against the recombinant protein.

### 2.4. Statistical Analysis

Results were presented as means ± standard deviations or counts and percentages. For quantitative features, the distributions were checked using the Shapiro-Wilk test. The Spearman rank correlation coefficient was used to study associations between gal-3 and echocardiographic parameters. The use of this test was dictated by the lack of normal distribution for the gal-3 variable. Statistical significance was set at *p* < 0.05. Statistical analyses were performed with STATISTICA 13.3 software (TIBCO Software Inc., Tulsa, OK, USA).

## 3. Results

The clinical and echocardiographic characteristics are presented in Table 1 and Table 2. The study included 63 patients scheduled for elective DCCV due to persistent AF. DCCV was successful in 43 (68.3%) patients and recovery of SR was achieved.

In our study group, all patients had developed left atrial structural remodeling expressed as LAVI > 34 mL/m^2^, assessed during AF before DCCV. The relatively low effectiveness of DCCV was caused by the selection of patients with left atrial structural remodeling.All examined patients had normal left ventricular contraction function. The characteristics of the study group show a tendency to elevated left ventricular filling pressure, assessed by measuring E/e’ and E/A in patients for whom DCCV was successful, and recovery of SR was achieved.

**Figure 1:**
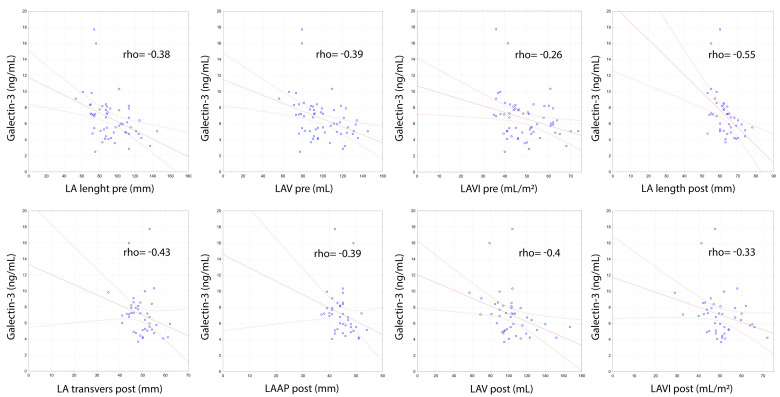
Correlations of galectin-3 concentration with echocardiographic measurements of left atrial dimensions and volume assessed before and after electrical cardioversion.

The analysis showed significant correlation of gal-3 level with the echocardiographic parameters assessing structure and function of the LA and LV (Table 3). Left atrial size, using echocardiographic parameters to assess dimensions and volume both before and after successful DCCV, was negatively correlated with gal-3 concentration. In the measurements of the size of the LA before DCCV, both the parameters assessing left atrial size (LA length pre, rho = −0.38; *p* = 0.003) and left atrial volume (LAV pre, rho = −0.39; *p* = 0.003; LAVI pre, rho = −0.26; *p* = 0.043; LAEDV pre, rho = −0.42; *p* = 0.002; LAESV pre, rho = −0.3; *p* = 0.028; LASV pre, rho = −0, 38; *p* = 0.005) were significantly negatively correlated with the concentration of gal-3 (Figure 1). These correlations were even more marked in patients for whom DCCV was effective, which allowed for the assessment of the left atrial size during SR. The assessment of the left atrial size during SR showed negative correlations in the dimensions of LAAP (post, rho = −0.39; *p* = 0.01), LA length (post, rho = −0.55; *p* < 0.001), LA transverse (post, rho = −0.43; *p* = 0.026) and left atrial volumes LAV (post, rho = −0.40; *p* = 0.008) and LAVI (post, rho = −0.33; *p* = 0.033) (Figure 1).

**Figure 2:**
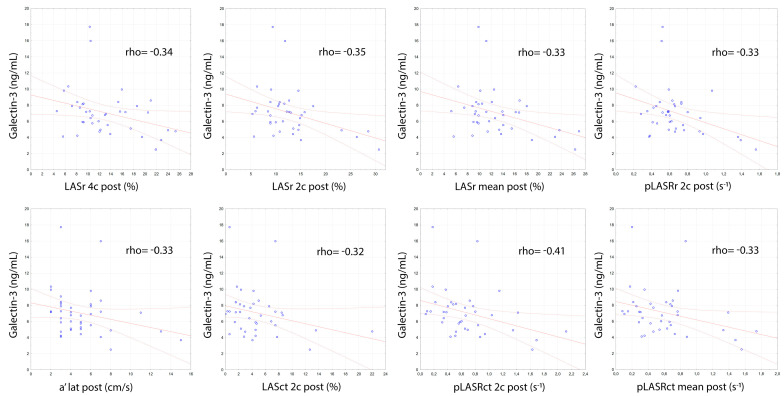
Correlations of galectin-3 concentration with echocardiographic measurements assessing left atrial compliance and contractility.

Left atria contractility assessed by two independent methods (i.e., tissue Doppler and by the assessment of left atrial strain and strain rate) was also negatively correlated with the gal-3 concentration. Left atrial contractility, assessed by tissue Doppler measuring the a’ lat post wave after successful DCCV, showed a negative correlation with the level of gal-3 (rho = −0.33; *p* = 0.033). Left atrial strain (LASct 2c post, rho = −0.32; *p* = 0.044) and strain rate in contraction phase assessed after successful DCCV were also negatively correlated with gal-3 levels (pLASRct 2c post, rho = −0.41; *p* = 0.01; pLASRct mean post, rho = −0.33; *p* = 0.038) (Figure 2).

**Figure 3:**
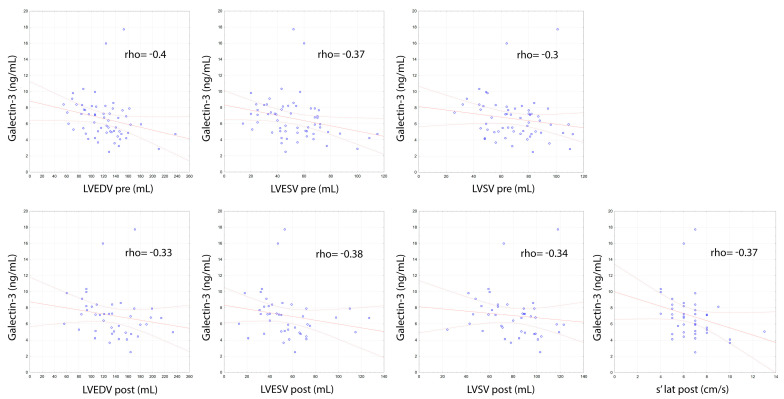
Correlations of galectin-3 concentration with echocardiographic measurements assessing left ventricular volume and contractility.

Left atrial compliance, assessed after successful DCCV using the left atrial strain technique, showed a negative correlation with gal-3 concentration (LASr 4c post, rho = −0.34; *p* = 0.032; LASr 2c post, rho = −0.35; *p* = 0.031; LASr mean post, rho = −0.33; *p* = 0.042; pLASRr 2c post, rho = −0.33; *p* = 0.042) (Figure 2). Negative correlations of gal-3 concentrations were also noted in relation to echocardiographic parameters assessing the size of the LV both before (LVEDV pre, rho = −0.4; *p* = 0.002; LVESV pre, rho = −0.37; *p* = 0.004; LVSV pre, rho = −0.3; *p* = 0.02) and after (LVEDV post, rho = −0.33; *p* = 0.032; LVESV post, rho = −0.38; *p* = 0.013; LVSV post, rho = −0.34; *p* = 0.027) effective DCCV and left ventricular contractility assessed by measuring the s’ lat post wave using tissue Doppler (rho = −0.37; *p* = 0.016) (Figure 3).

## 4. Discussion

In our study, we showed that gal-3 level negatively correlates with the size of the LA cavity, one of the main echocardiographic parameters assessing atrial structural remodeling. In our group, patients with higher concentrations of gal-3 had a smaller-sized LA, which suggests that left atrial fibrosis may lead to a reduction in its dimensions and volume. This correlation was visible in the left atrial measurements taken during AF and in the measurements obtained after the SR had been restored following successful DCCV. In addition, we demonstrated that gal-3 concentration is negatively correlated with the left atrial reservoir strain assessed during SR, a parameter that assesses left atrial compliance. It follows that the higher the concentration of the fibrosis marker, the lower the left atrial compliance. This may indicate a decrease in the number of stretchable components, i.e., muscle tissue, and an increase in non-stretchable components, i.e., fibrous tissue in the left atrial wall. Negative correlations of gal-3 levels with contractility strain and the a’ wave of the lateral part of the mitral annulus assessed during SR after effective DCCV, which were the parameters assessing the left atrial contractility, also indicate the reduction of muscle tissue in the left atrial wall. These data suggest that the high concentration of gal-3 in patients with AF may indirectly indicate advanced left atrial fibrosis.

So far, the assessment of left atrial structural remodeling has been based mainly on assessing the dimensions of this heart cavity via echocardiographic examination. The results of our study suggest that gal-3 could be used to assess the left atrial structural remodeling at the cellular level.

In the studied group of patients, we also noted a negative correlation of gal-3 level with the volume of the LV, which additionally suggests that fibrosis may lead to a reduction in the dimensions of the LV, which is also emphasized by the observation of the effect of gal-3 concentration on the reduction of dimensions and volume of the LA. A negative correlation of gal-3 concentration was also noted when measuring the s’ wave of the lateral part of the mitral annulus, which indicates that fibrosis may reduce the left ventricular contractility in the same way as we described it in relation to the left atrial contractility. There is extensive literature on galectins as particles that play a key role in chronic inflammation, apoptosis, immune response, cancer, atherosclerosis, microbial infections, leukemia, multiple myeloma, pulmonary fibrosis, and viral infections [33]. Reports regarding the role of gal-3 in AF are contradictory [31,34]. Gurses et al. showed that the concentration of gal-3 has a prognostic value for maintaining SR after DCCV is performed in patients with persistent AF and who received amiodarone treatment before DCCV [25]. In contrast, Begg et al. showed that gal-3 concentration is not prognostic for maintaining SR after DCCV [22]. Similarly, Merino-Merino et al. showed that gal-3 concentration measured at the time of DCCV and 6 months after DCCV is not prognostic for maintaining sinus rhythm after DCCV [39]. In the group of patients with persistent AF studied by our team, we also did not show that the level of gal-3 was prognostic in terms of maintaining SR after DCCV [28]. It should be noted that DCCV does not remove the substrate of arrhythmia, which may be left atrial remodeling, but only terminates the arrhythmia episode. Ablation is a method of treating AF that can remove the arrhythmia substrate. In the case of AF ablation and the prognostic value of gal-3 in maintaining SR, the available reports are also inconsistent. Gal-3 has been shown to be prognostic for maintaining SR after ablation in patients with persistent arrhythmia without structural heart disease and in groups of patients with paroxysmal and persistent arrhythmias [24,29]. In contrast to these findings, there are reports by Celik et al., Kornej et al. and Begg et al., in which the concentration of gal-3 has not been shown to have a prognostic value in terms of maintaining SR after AF ablation [23,27,32]. In the meta-analysis by Zhang et al., which included 7 studies with patients with AF who underwent ablation, it was shown that patients with recurrent AF had a higher concentration of gal-3 before ablation than patients who maintained SR [31]. Gurses et al. showed that the level of gal-3 is higher in patients with persistent AF than in patients with paroxysmal arrhythmia. They also showed a significant positive correlation of gal-3 level with LAVI, which contradicts the results presented by our team [26]. Similarly, Wang et al. demonstrated that patients with elevated gal-3 levels have a higher risk of AF progressing from paroxysmal to persistent form [40]. In the ARIC study, patients who had a higher concentration of gal-3 were significantly more likely to be diagnosed with AF, although this significance decreased when corrected for the presence of ischemic heart disease or heart failure [41]. Kornej et al. emphasize that the concentration of gal-3 in patients with AF is correlated with BMI and not with the severity of AF or the prognosis for maintaining SR after ablation [27]. The study by Ansari et al. showed a positive correlation between the concentration of gal-3 and the LA area and the anteroposterior dimension of LA, but in this study only 13% of patients were diagnosed with persistent AF. This study showed that the concentration of gal-3 has a diagnostic value in terms of detecting severe grade left ventricular diastolic dysfunction. The results of the study by Ansari et al. are inconsistent with those presented by us in terms of the correlation of gal-3 concentration and LA size. This discrepancy may result from the selection of the test group. In our study, all patients had persistent AF and the selection of patients was not dictated by the diagnosis of left ventricular diastolic dysfunction. Additionally, the discrepancies in the results may be due to various causes of LA wall fibrosis; in our study this was AF, while in the study by Ansari et al. it was elevated pressure in the LA cavity and the associated LA dilatation due to left ventricular diastolic dysfunction [42]. The negative correlation of gal-3 concentration with the parameters assessing the size of the LA, which would indicate the possibility of a reduction of dimensions and volume of the left atrial cavity due to fibrosis, partly contradicts current reports. Some authors state that the correlations between the size of the LA and the concentration of gal-3 in their study groups were significantly positive, and some say that no such correlation was found [43,44,45]. The relationship between gal-3 concentration and fibrosis of the left atrial or right appendage is also inconsistent in the available literature [30,44,45]. Even though many studies have found gal-3 to be associated with remodeling of the LA and left atrial appendage, the study by Berger et al. showed no correlation between the concentration of gal-3 in the blood serum and the left atrial appendage wall, and the fibrosis of the left atrial appendage. There was also no difference in the content of fibrous tissue in the left atrial appendage between patients with paroxysmal and persistent AF. Nor did this study show that the concentration of gal-3 measured before ablation was prognostic in terms of maintaining SR. In the study by Berger et al., only an increase in gal-3 concentration measured 6 months after ablation had prognostic value in terms of the maintenance of SR after surgical ablation, and patients with increased gal-3 concentration had a higher content of thick collagen strands in the left atrial appendage than patients in whom the concentration of gal-3 decreased or remained unchanged [45]. In contrast, Hernández-Romero et al. showed that serum gal-3 concentration correlates with interstitial fibrosis measured in the right atrial appendage as obtained by resection [44]. In contrast to the study by Berger et al., Tang et al.’s study found that gal-3 concentration was associated with left atrial appendage remodeling assessed by measuring the velocity of left atrial appendage emptying. This study showed that high gal-3 concentration is associated with a higher risk of thrombus formation in the left atrial appendage cavity, which is also an indirect symptom of left atrial remodeling [46]. In addition, in the study by Yalcin et al., the concentration of gal-3 was significantly correlated with left atrial fibrosis assessed by magnetic resonance imaging in patients with AF [30].

### Study Limitations

Our study was single center and performed on a small group of patients. The data on AF duration were obtained retrospectively from patient reports. When interpreting our results, one should remember that echocardiography is a subjective method that is highly operator-dependent and requires experience and skill. Therefore, in our study, all echocardiographic examinations were performed by one experienced operator. All DCCV procedures were performed in the antero-lateral position, without changing the paddle position in the case of DCCV failure. This might have influenced the success rate. We did not perform control measurements of gal-3 in patients whose SR was maintained; consequently, we cannot assess the diagnostic value of gal-3 for reverse remodeling.

## 5. Conclusions

The concentration of gal-3 significantly negatively correlates with the size, systolic function, and compliance of the left atrial wall in patients with persistent AF. The concentration of gal-3 negatively correlates with the size and systolic function of the LV in patients with persistent AF. The assessment of gal-3 concentration in patients with persistent AF may help in the assessment of left atrial remodeling.

Patients who will be treated with galectin inhibitors should be monitored echo-cardio-graphically for changes in the size and function of the LA and LV. Further studies should assess the usefulness of fibrosis markers in predicting outcomes in patients with AF.

## Figures and Tables

**Table 1:** Clinical Characteristics.

	N	$\bar{x}$ ± sd or n (%)
Age (years)	63	64.92 ± 8.59
Age < 65, n (%)	63	28 (44.4)
Age 65–74, n (%)	63	26 (41.3)
Age ≥ 75, n (%)	63	9 (14.3)
Males, n (%)	63	42 (66.7)
BMI (kg/m^2^)	63	30.42 ± 4.73
Smokers, n (%)	63	6 (14.29)
Total AF duration (months)	56	26.68 ± 38.46
AF duration current episode (weeks)	56	15.28 ± 23.66
Number of previous DCCV	63	0.41 ± 0.84
Hypertension, n (%)	63	56 (88.9)
Diabetes mellitus, n (%)	63	13 (20.6)
Stroke/TIA, n (%)	63	8 (12.7)
CHA_2_DS_2_-VASC	63	2.63 ± 1.42
HAS-BLED	63	0.52 ± 0.69
Coronary artery disease, n (%)	63	10 (15.87)
GFR, (mL/min)	63	65.82 ± 16.77
Chronic kidney disease, stage (mean)	63	2.24 ± 0.69
Obstructive pulmonary disease, n (%)	63	3 (4.8)
Amiodarone pre, n (%)	63	6 (9.5)
Beta-blockers pre, n (%)	63	57 (60.5)
Calcium channel blockers pre, n (%)	63	11 (17.5)
ACE inhibitors/ARB pre, n (%)	63	55 (87.3)
Statins pre, n (%)	63	40 (63.5)
Diuretics pre, n (%)	63	25 (39.7)
Spironolactone/eplerenone pre, n (%)	63	7 (11.1)
Galectin-3 (ng/mL)	63	6.59 ± 2.61
Creatinine (mg/dL)	63	1.16 ± 0.21
Troponin T (ng/L)	63	10.18 ± 9.16
BNP (pg/mL)	43	172.77 ± 167.39
CRP (mg/L)	59	7.12 ± 21.35

Key to Acronyms and Abbreviations: ACE inhibitors/ARB, angiotensin-converting enzyme inhibitors/angiotensin II receptor blockers; AF, atrial fibrillation; BMI, body mass index; BNP, B-type natriuretic peptide; CRP, C-reactive protein; DCCV, direct current cardioversion; GFR, glomerular filtration rate; post, after cardioversion; pre, before cardioversion; n (%), counts and percentages; TIA, transient ischemic attack; $\bar{x}$ ± sd, means ± standard deviations.

**Table 2:** Echocardiographic Characteristics.

	N	$\bar{x}$ ± sd
RV pre (mm)	59	31.39 ± 3.80
IVS pre (mm)	59	10.68 ± 1.51
LVEDD pre (mm)	59	51.17 ± 6.18
LVESD pre (mm)	59	33.71 ± 7.20
RVSP pre (mmHg)	55	25.05 ± 11.89
LAAP pre (mm)	59	43.54 ± 4.76
LA length pre (mm)	59	62.03 ± 5.18
LA transverse pre (mm)	59	48.25 ± 5.20
LAV pre (mL)	59	98.80 ± 20.34
LAVI pre (mL/m^2^)	59	49.42 ± 9.71
LAEDV Index pre (mL/m^2^)	59	36.13 ± 9.22
LAEF pre (%)	59	27.26 ± 11.60
LAEDV pre (mL)	55	93.24 ± 19.93
LASV pre (mL)	55	26.57 ± 10.90
RAA s pre (cm^2^)	59	23.16 ± 4.94
RAA d pre (cm^2^)	59	17.07 ± 4.05
LVEDV pre (mL)	59	121.24 ± 35.33
LVESV pre (mL)	59	52.31 ± 21.39
LVSV pre (mL)	59	69.53 ± 20.08
LVEF pre (%)	59	58.75 ± 9.96
s’ lat pre (cm/s)	59	6.34 ± 1.64
e’ lat pre (cm/s)	59	11.75 ± 3.03
s’ mid pre (cm/s)	59	4.93 ± 0.98
e’ mid pre (cm/s)	59	7.97 ± 1.84
s’ mean pre (cm/s)	59	5.65 ± 1.21
e’ mean pre (cm/s)	59	9.86 ± 2.19
E pre (m/s)	59	0.82 ± 0.19
LAAP post (mm)	42	44.71 ± 4.08
LA length post (mm)	43	62.95 ± 5.95
LA transverse post (mm)	43	49.40 ± 5.39
LAV post (mL)	43	102.67 ± 20.54
LAVI post (mL/m^2^)	43	50.32 ± 8.45
LAEDV Index post (mL/m^2^)	42	32.62 ± 9.49
LAEF post (%)	42	34.83 ± 12.04
LAEDV post (mL)	39	92.51 ± 16.69
LASV post (mL)	38	31.58 ± 10.35
RAA s post (cm^2^)	41	24.31 ± 5.52
RAA d post (cm^2^)	41	15.78 ± 4.13
LVEDV post (mL)	42	134.38 ± 41.22
LVESV post (mL)	42	54.86 ± 24.59
LVSV post (mL)	42	79.62 ± 23.26
LVEF post (%)	42	61.00 ± 8.60
s’ lat post (cm/s)	42	6.61 ± 1.76
e’ lat post (cm/s)	42	10.32 ± 2.87
a’ lat post (cm/s)	42	5.02 ± 2.83
s’ mid post (cm/s)	41	5.68 ± 1.39
e’ mid post (cm/s)	41	7.40 ± 1.95
a’ mid post (cm/s)	41	5.29 ± 2.35
s’ mean post (cm/s)	41	6.17 ± 1.40
e’ mean post (cm/s)	41	8.82 ± 2.21
a’ mean post (cm/s)	41	5.22 ± 2.48
E/e’ lat ratio post	41	9.06 ± 3.81
E/e’ mid ratio post	40	12.41 ± 4.83
E/e’ mean ratio post	40	10.36 ± 4.19
E post (m/s)	42	0.85 ± 0.20
A post (m/s)	42	0.39 ± 0.16
E/A ratio post	42	2.60 ± 1.21
E DT post (ms)	46	177.29 ± 42.11
LASr 4c pre (%)	57	8.82 ± 3.34
pLASRr 4c pre (s^−1^)	54	0.52 ± 0.24
pLASRcd 4c pre (s^−1^)	54	0.94 ± 0.30
LASr 2c pre (%)	54	8.56 ± 4.14
pLASRr 2c pre (s^−1^)	51	0.59 ± 0.24
pLASRcd 2c pre (s^−1^)	51	0.91 ± 0.36
LASr mean pre (%)	54	8.69 ± 3.27
pLASRr mean pre (s^−1^)	51	0.56 ± 0.21
pLASRcd mean pre (s^−1^)	51	0.92 ± 0.29
LASr 4c post (%)	39	13.02 ± 5.45
LAScd 4c post (%)	39	9.07 ± 3.59
LASct 4c post (%)	39	3.96 ± 3.48
pLASRr 4c post (s^−1^)	39	0.66 ± 0.24
pLASRcd 4c post (s^−1^)	39	0.82 ± 0.29
pLASRct 4c post (s^−1^)	39	0.54 ± 0.39
LASr 2c post (%)	39	12.75 ± 5.85
LAScd 2c post (%)	39	8.00 ± 3.69
LASct 2c post (%)	39	4.74 ± 4.26
pLASRr 2c post (s^−1^)	39	0.67 ± 0.29
pLASRcd 2c post (s^−1^)	39	0.75 ± 0.34
pLASRct 2c post (s^−1^)	39	0.68 ± 0.46
LASr mean post (%)	39	12.89 ± 5.25
LAScd mean post (%)	39	8.54 ± 3.02
LASct mean post (%)	39	4.35 ± 3.66
pLASRr mean post (s^−1^)	39	0.66 ± 0.24
pLASRcd mean post (s^−1^)	39	0.79 ± 0.26
pLASRct mean post (s^−1^)	39	0.61 ± 0.41

Key to Acronyms and Abbreviations: 2c, two-chamber projection; 4c, four-chamber projection; A wave, late mitral inflow wave; a’ wave, late diastolic mitral annular velocity; cd, conduit phase; ct, contractile phase; d, diastolic; DCCV, direct current cardioversion; E, early filling wave; e’, early diastolic mitral annular velocity; E DT, deceleration time of E wave; IVS, intraventricular septum wall thickness; LA, left atrium; LAAP, left atrial antero-posterior diameter; LAEDV, left atrial end-diastolic volume; LAEF, left atrial emptying fraction; LAS, left atrial strain; LASV, left atrial stroke volume; lat, measurements obtained from the lateral part of the mitral ring; LAV, left atrial volume; LAVI, left atrial volume index; LVEDD, left ventricular end-diastolic diameter; LVEDV, left ventricular end-diastolic volume; LVEF, left ventricular ejection fraction; LVESD, left ventricular end-systolic diameter; LVESV, left ventricular end-systolic volume; LVSV, left ventricular stroke volume; mid, measurements obtained from the medial part of the mitral ring; n (%), counts and percentages; pLASR, peak left atrial strain rate; post, measurements obtained after successful cardioversion; pre, measurements obtained before cardioversion; r, reservoir phase; RAA, right atrium area; RV, right ventricular; RVSP, right ventricular systolic pressure; s’ wave, systolic mitral annular velocity; s, systolic; $\bar{x}$ ± sd, means ± standard deviations.

**Table 3:** Correlations of galectin-3 concentration with the assessed echocardiographic parameters.

	rho	95% CI	*p*
RV pre (mm)	−0.20	−0.06–0.43	0.127
IVS pre (mm)	0.04	−0.22–0.29	0.758
LVEDD pre (mm)	−0.21	−0.44–0.05	0.113
LVESD pre (mm)	−0.24	−0.46–0.02	0.067
RVSP pre (mmHg)	0.03	−0.24–0.29	0.820
LAAP pre (mm)	−0.12	−0.36–0.14	0.385
LA length pre (mm)	−0.38	−0.58–−0.14	0.003
LA transverse pre (mm)	−0.18	−0.41–0.08	0.185
LAV pre (mL)	−0.39	−0.58–−0.15	0.003
LAVI pre (mL/m^2^)	−0.26	−0.48–0.00	0.043
LAEDV Index pre (mL/m^2^)	−0.18	−0.41–0.08	0.164
LAEF pre (%)	−0.11	−0.35–0.15	0.389
LAEDV pre (mL)	−0.42	−0.61–−0.17	0.002
LASV pre (mL)	−0.38	−0.58–−0.13	0.005
RAA s pre (cm^2^)	−0.10	−0.35–0.17	0.462
RAA d pre (cm^2^)	−0.06	−0.31–0.20	0.626
LVEDV pre (mL)	−0.40	−0.59–−0.16	0.002
LVESV pre (mL)	−0.37	−0.57–−0.12	0.004
LVSV pre (mL)	−0.30	−0.51–−0.05	0.020
LVEF pre (%)	0.10	−0.34–0.16	0.456
s’ lat pre (cm/s)	−0.15	−0.39–0.11	0.266
e’ lat pre (cm/s)	−0.17	−0.41–0.09	0.208
s’ mid pre (cm/s)	−0.08	−0.33–0.18	0.540
e’ mid pre (cm/s)	0.04	−0.22–0.29	0.790
s’ mean pre (cm/s)	−0.16	−0.40–0.10	0.227
e’ mean pre (cm/s)	−0.12	−0.36–0.14	0.349
E pre (m/s)	0.08	−0.18–0.33	0.567
LAAP post (mm)	−0.39	−0.62–−0.10	0.010
LA length post (mm)	−0.55	−0.73–−0.30	0.000
LA transverse post (mm)	−0.43	−0.64–−0.15	0.026
LAV post (mL)	−0.40	0.062–−0.11	0.008
LAVI post (mL/m^2^)	−0.33	−0.57–−0.03	0.033
LAEDV Index post (mL/m^2^)	0.08	−0.23–0.37	0.595
LAEF post (%)	−0.10	−0.39–0.21	0.525
LAEDV post (mL)	−0.20	−0.48–0.12	0.212
LASV post (mL)	−0.25	−0.52–0.07	0.124
RAA s post (cm^2^)	0.05	−0.26–0.35	0.752
RAA d post (cm^2^)	−0.01	−0.31–0.29	0.934
LVEDV post (mL)	−0.33	−0.57–−0.03	0.032
LVESV post (mL)	−0.38	−0.61–−0.09	0.013
LVSV post (mL)	−0.34	−0.58–−0.04	0.027
LVEF post (%)	0.09	−0.22–0.38	0.585
s’ lat post (cm/s)	−0.37	−0.60–0.07	0.016
e’ lat post (cm/s)	−0.02	−0.32–0.28	0.907
a’ lat post (cm/s)	−0.33	−0.57–−0.03	0.033
s’ mid post (cm/s)	−0.24	−0.50–0.07	0.135
e’ mid post (cm/s)	−0.14	−0.24–0.17	0.400
a’ mid post (cm/s)	−0.24	−0.50–0.07	0.139
s’ mean post (cm/s)	−0.31	−0.56–−0.003	0.050
e’ mean post (cm/s)	−0.06	−0.35–0.25	0.724
a’ mean post (cm/s)	−0.25	−0.51–0.06	0.114
E/e’ lat ratio post	0.03	−0.28–0.33	0.854
E/e’ mid ratio post	0.19	−0.13–0.47	0.249
E/e’ mean ratio post	0.16	−0.16–0.44	0.321
E post (m/s)	0.11	−0.20–0.40	0.504
A post (m/s)	−0.21	−0.48–0.10	0.175
E/A ratio post	0.17	−0.14–0.45	0.272
E DT post (ms)	0.03	−0.27–0.33	0.850
LASr 4c pre (%)	−0.09	−0.34–0.17	0.528
pLASRr 4c pre (s^−1^)	−0.04	−0.30–0.23	0.767
pLASRcd 4c pre (s^−1^)	−0.20	−0.44–0.07	0.142
LASr 2c pre (%)	−0.19	−0.43–0.08	0.161
pLASRr 2c pre (s^−1^)	0.01	−0.26–0.28	0.921
pLASRcd 2c pre (s^−1^)	−0.25	−0.49–0.03	0.078
LASr mean pre (%)	−0.17	−0.41–0.10	0.208
pLASRr mean pre (s^−1^)	−0.09	−0.35–0.19	0.537
pLASRcd mean pre (s^−1^)	−0.28	−0.51–−0.005	0.047
LASr 4c post (%)	−0.34	−0.59–−0.03	0.032
LAScd 4c post (%)	−0.25	−0.52–0.07	0.131
LASct 4c post (%)	−0.22	−0.50–0.10	0.176
pLASRr 4c post (s^−1^)	−0.15	−0.44–0.17	0.377
pLASRcd 4c post (s^−1^)	−0.24	−0.51–0.08	0.138
pLASRct 4c post (s^−1^)	−0.28	−0.54–0.04	0.090
LASr 2c post (%)	−0.35	−0.59 –−0.04	0.031
LAScd 2c post (%)	−0.19	−0.47–0.13	0.258
LASct 2c post (%)	−0.32	−0.57–−0.01	0.044
pLASRr 2c post (s^−1^)	−0.33	−0.58–−0.02	0.042
pLASRcd 2c post (s^−1^)	−0.23	−0.50–0.09	0.168
pLASRct 2c post (s^−1^)	−0.41	−0.64–−0.11	0.010
LASr mean post (%)	−0.33	−0.58–−0.02	0.042
LAScd mean post (%)	−0.30	−0.56–0.02	0.064
LASct mean post (%)	−0.30	−0.56–0.02	0.068
pLASRr mean post (s^−1^)	−0.25	−0.52–0.07	0.121
pLASRcd mean post (s^−1^)	−0.24	−0.51–−0.08	0.136
pLASRct mean post (s^−1^)	−0.33	−0.58–−0.02	0.038

Key to Acronyms and Abbreviations found with Table 2.

## Data Availability

The data underlying this article will be shared on reasonable request to the corresponding author.
